# Review of the India Adolescent Health Strategy in the context of disease burden among adolescents

**DOI:** 10.1016/j.lansea.2023.100283

**Published:** 2023-09-28

**Authors:** Rakhi Dandona, Anamika Pandey, G Anil Kumar, Monika Arora, Lalit Dandona

**Affiliations:** a Public Health Foundation of India, New Delhi, India; b Institute for Health Metrics and Evaluation, University of Washington, Seattle, USA

**Keywords:** Adolescent, Disease burden, Gender, Global Burden of Disease Study, Health Management Information System, India, Injuries, Policy, Program, Service provision, Strategy

## Abstract

**Background:**

A nuanced understanding of the health needs of adolescents in the context of the India Adolescent Health Strategy (IAHS) is needed to inform policy interventions for improving the health and well-being of adolescents in India.

**Methods:**

Using data from the Global Burden of Diseases, Injuries, and Risk Factors Study 2019, we identified the top ten causes of years of life lost (YLLs), years lived with disability (YLDs), and disability-adjusted life years (DALYs) disaggregated by sex and age group (10–14 and 15–19 years) for India and its states in 2019. To inform the IAHS of refinement or expansion in focus needed to improve adolescent health in India, we reviewed the extent to which the top 10 causes of disease burden are addressed in the IAHS, and the availability of and age- and sex-disaggregation in the service utilisation data for adolescents captured in the Adolescent Friendly Health Clinic monitoring information system (AFHC MIS) and Health Management Information System (HMIS). We also reviewed the availability of and age-and sex-disaggregation in the data capture at the population level for the IAHS outcome indicators in the data sources identified in the IAHS operational framework.

**Findings:**

Females in the 10–14 and 15–19 years age groups suffered 6.75 million and 9.25 million DALYs, respectively, 39.1% and 44.2% of which were YLLs; the corresponding DALYs for males were 6.71 million and 9.65 million (42.3% and 41.1% YLLs), respectively. Within the 6 thematic areas of the IAHS, most strategies and indicators identified are for sexual and reproductive health followed by nutrition, and broadly these conditions accounted for YLDs and not YLLs in adolescents. Significant gaps in the IAHS in comparison to the disease burden for fatal diseases and conditions were seen across injuries, communicable diseases, and non-communicable diseases. Injuries accounted for 65.9% and 45.3% of YLLs in males and females aged 15–19 years, and 40.8% in males aged 10–14 years. Specifically, road injuries (15.3%, 95% UI 11.0–18.0) and self-harm (11.3%, 95% UI 8.7–14.2) accounted for most of the injury deaths in 15–19 years whereas drowning (7.7% 95% UI 5.8–9.6) and road injuries (6.9%, 95% UI 4.7–8.6) accounted for the most injury deaths in 10–14 years males. However, only self-harm and gender-based violence are specifically addressed in the IAHS with non-specific interventions for other injuries. Diarrhoea, lower respiratory infections, malaria, encephalitis, tuberculosis, typhoid, cirrhosis, and hepatitis are the other disease conditions accounting for YLLs and DALYs in adolescents but these are neither addressed in the IAHS nor in service provision under the AFHC MIS. There is no age- or sex-disaggregation in the cause of death data captured in the HMIS to allow an understanding of mortality in adolescents. For the IAHS outcome indicators at the population level, data capture for the 10–14 years irrespective of sex was largely missing from the population surveys and none of the surveys captured data for either females or males aged 15–19 years for physical inactivity and mental health indicators.

**Interpretation:**

The considerable differences seen in the IAHS thematic focus as compared with the leading causes of fatal and non-fatal disease burden in adolescents in India, and in the availability of population-level data to monitor the outcome indicators of the IAHS can pose substantial limitations for improving adolescent health in India. The findings in this paper can be utilized by decision makers to refine action aimed at improving adolescent health and well-being.

**Funding:**

10.13039/100000865Bill & Melinda Gates Foundation.

**Research in context**

**Evidence before this study**

The India Adolescent Health Strategy (IAHS) was launched in 2014. We searched PubMed and publicly available reports using the search terms “adolescent”, “health”, “India”, “mortality”, and “programming” on 31 March 2023 published in the last 10 years without language restrictions. We found a variety of publications documenting disease-specific issues in adolescents including anaemia, reproductive health, mental health, obesity, tobacco use, and on programming and rapid reviews of school health programs. One rapid review on adolescent health programming documented the system-related issues in implementing the program, and another assessed the policy environment for addressing adolescent mental health in India. No publication was found documenting the disease burden in adolescents in India and assessing the age- and sex-disaggregated coverage of the diseases/conditions, their service coverage, and outcome indicators under the IAHS.

**Added value of this study**

The IAHS specifically addresses sexual and reproductive health and nutrition. Significant gaps in IAHS are seen for injuries/violence despite road injuries, self-harm and drowning accounting for the majority of the fatal burden in the adolescents. Only self-harm and gender-based violence are covered in the IAHS along with the prevention of non-specific injuries. Diarrhoea, lower respiratory infections, malaria, encephalitis, tuberculosis, typhoid, cirrhosis, and hepatitis are the other disease conditions accounting for disease burden in adolescents but are not covered under the IAHS, and service provision indicators for many are not tracked. For the IAHS outcome indicators at the population level, data capture for 10–14 years irrespective of sex was largely missing from the population surveys that are used to track the IAHS indicators, and none of the surveys captured data for either females or males aged 15–19 years for physical inactivity and mental health indicators.

**Implications of all the available evidence**

Many of the top ten leading causes of fatal and non-fatal disease burden in adolescents in India are currently not covered under the IAHS. With adolescents constituting about one-fifth of India's population and young people, these findings can facilitate action by age, sex and state from the decision makers to address adolescent health and well-being more comprehensively.

## Introduction

Commitment towards child and adolescent health has gained momentum globally and in India in the past decade.^1^, ^2^, ^3^, ^4^ Adolescents (10–19 years), estimated at 268 million in 2019, constitute about one-fifth of India's population and young people.^5^ The large and increasing relative share and absolute numbers of the adolescent population in India make it necessary to address their health and development needs to achieve improved health and development outcomes for the population as a whole. To this effect, the India Adolescent Health Strategy (IAHS) is based on the principles of adolescent leadership and participation, human rights, equity and inclusion, gender equity, and strategic partnerships. This strategy envisions, that all adolescents in India are able to realize their full potential by making informed and responsible decisions related to their health and well-being and by accessing the services and support they need to do so.^4^

The IAHS operational framework is organised under 6 themes which are the content of the programme and serve as the broad action areas of the strategic framework; each theme, in turn, prioritises one or more goals, to be addressed by strategies and interventions, and measured by indicators (Panel 1).^4^ We undertook a review of the 6 themes of the IAHS in the context of the top 10 causes of disease burden in this age group, as estimated by the Global Burden of Disease (GBD) Study, disaggregated by age and sex to assess if these are addressed in the IAHS. Further, we reviewed the availability of and age-and sex-disaggregation in the data capture at the population level for the outcome indicators of the IAHS in the data sources identified in the operational framework of this strategy,^6^ and lastly, we assessed the availability of age- and sex-disaggregation in the service utilisation data for adolescents. As the premise of this report is to inform the IAHS of expansion in focus to improve adolescent health in India, we have utilised the most recent disease burden data for it to be relevant for the adolescent program at this time for action.

*Panel 1* **Themes and priorities of the India Adolescent Health Strategy.**

**1. Improve Nutrition**

• Reduce the prevalence of malnutrition in adolescent girls and boys (including overweight/obesity)
• Reduce the prevalence of iron deficiency anaemia among adolescent girls and boys

**2. Enable sexual and reproductive health (SRH)**

• Improve knowledge, attitudes and behaviour, in relation to SRH
• Reduce teenage pregnancies
• Improve birth preparedness, complication readiness, and provide early parenting support for adolescent parents

**3. Enhance mental health**

• Address mental health concerns of adolescents

**4. Prevent injuries and violence**

• Promote favourable attitudes for preventing injuries and violence (including gender-based violence) among adolescents

**5. Prevent substance misuse**

• Increase adolescents' awareness of the adverse effects and consequences of substance misuse

**6. Address conditions for non-communicable diseases (NCDs)**

• Promote behaviour change in adolescents to prevent NCDs such as cancer, diabetes, cardio-vascular diseases, and stroke

## Methods

We compared the definitions of adolescent age groups used in India and globally.^7^, ^8^, ^9^ Adolescents are defined in India as individuals in the 10–19 years age group, which is consistent with the global definition. As the disease burden differs between 10 and 14 years and 15–19 years,^3^^,^^10^ we present the findings disaggregated by these two age groups–10–14 years and 15–19 years—to provide a more nuanced understanding of adolescent health needs to identify and act upon the differential needs of these two age groups. The GBD also provides data for adolescents in these two age groups only. We accessed the GBD 2019 data from the Institute of Health Metrics and Evaluation's Global Health Data Exchange on 30th November 2022.^11^ The accessed data were the absolute number and count per 100,000 people for years of life lost (YLLs), years lived with disability (YLDs), and disease burden measured as disability-adjusted life years (DALYs) for females and males aged 10–14 years and 15–19 years for India and its states in 2019 for causes relating to communicable, maternal, neonatal, and nutritional diseases (CMNNDs), non-communicable diseases (NCDs) and injuries. A comprehensive description of the metrics, data sources, and statistical modelling for disease burden and risk factor estimation in GBD 2019 has been provided elsewhere.^12^, ^13^, ^14^ The GBD complies with the Guidelines for Accurate and Transparent Health Estimates Reporting (GATHER) statement. The GBD 2019 methods relevant to this report are summarised here. YLLs were computed from observed deaths and reference standard life expectancy at the age of death, which was obtained from the GBD standard life table.^13^ The major data sources used for cause-specific mortality estimation in India were verbal autopsy from Sample Registration System, Medically Certified Causes of Death, cancer registries, and smaller verbal autopsy studies.^15^ The quality and comparability of the cause of death data are assessed and enhanced through multiple steps in GBD as reported previously.^12^^,^^13^^,^^15^ The major input data sources used to quantify the non-fatal burden of disease in India were representative population-level surveys and cohort studies, programme-level data on disease burden from government agencies, surveillance system data on disease burden, administrative records of health-service encounters, disease registries, and a wide range of other studies done across India.^15^ These studies included published literature as well as unpublished studies that were identified and accessed through a network of expert group members and collaborators in India. YLDs were estimated as the product of prevalence estimate and a disability weight for health states of each mutually exclusive sequela adjusted for comorbidities. DALYs, a summary measure of total health loss, were computed for India and states by summing YLLs and YLDs for each cause, age, and sex.^12^

We present the top 10 individual causes of DALYs, YLLs and YLDs disaggregated by sex and by two age groups as provided by GBD to highlight the differential fatal and non-fatal burden on adolescent health for India in 2019 from the GBD. The percentage contribution of each of the top 10 individual causes to all-cause DALYs, YLLs and YLDs is presented. We also report on the contribution of major disease groups to total DALYs, YLLs and YLDs from the top 10 causes due to CMNNDs, NCDs and injuries disaggregated by sex and age group in India in 2019. In addition, we report on the sub-national heterogeneity in the top 10 causes of DALYs for the two age groups by sex in 2019. These findings are reported for 31 geographical units in India: the 28 states, the union territory of Delhi, the union territories of Jammu & Kashmir and Ladakh (combined), and the other smaller union territories combined (Andaman and Nicobar Islands, Chandigarh, Dadra and Nagar Haveli, Daman and Diu, Lakshadweep, and Puducherry). We report all the estimates with 95% uncertainty intervals (UIs) where relevant. UIs were based on 1000 runs of the models for each quantity of interest, with the mean regarded as the point estimate and the 2.5th and 97.5th percentiles considered the 95% UI. The accessed data was downloaded in CSV format as available from Global Health Data Exchange.^11^ The data from CSV files were copied in MS Excel to generate the proportions and estimate the percent change presented in this paper.

We undertook a review of the IAHS to assess if the strategy catered to the top 10 individual causes of disease burden identified by the GBD among adolescent females and males aged 10–14 years and 15–19 years. Service delivery data for adolescents are available from the Adolescent Friendly Health Clinic Management Information System (AFHC MIS),^16^ and from the Health Management Information System (HMIS) to monitor service delivery under various national health programmes predominately in the public sector health facilities.^17^ We assessed the age- and sex-disaggregation in service delivery indicators in the AFHC MIS and HMIS on adolescent health. In addition, the IAHS operational framework has also identified data sources at the population level to monitor outcome indicators, which include National Family Health Survey (NFHS), District Level Household & Facility Survey (DLHS), and Annual Health Survey (AHS). We reviewed the availability and the level of age- and sex-disaggregation in the data capture of these indicators in the relevant study tools from the recent rounds of NFHS-5 (2019–2021), DLHS-4 (2014–2016), and AHS baseline and 2nd update surveys (2010–11 and 2012–13). The household, woman, man, and biomarker questionnaires were reviewed for NFHS-5,^18^, ^19^, ^20^, ^21^ the household and woman questionnaires for DLHS-4,^22^^,^^23^ and household, woman, mortality, and bio-marker schedules were reviewed for AHS.^24^, ^25^, ^26^, ^27^, ^28^ The IAHS operational framework also refers to ongoing rapid assessments of nutritional and health outcomes among adolescents, however, no further information was readily available about these assessments in the public domain.^6^

## Results

### Disease burden among female adolescents

**Fig. 1:**
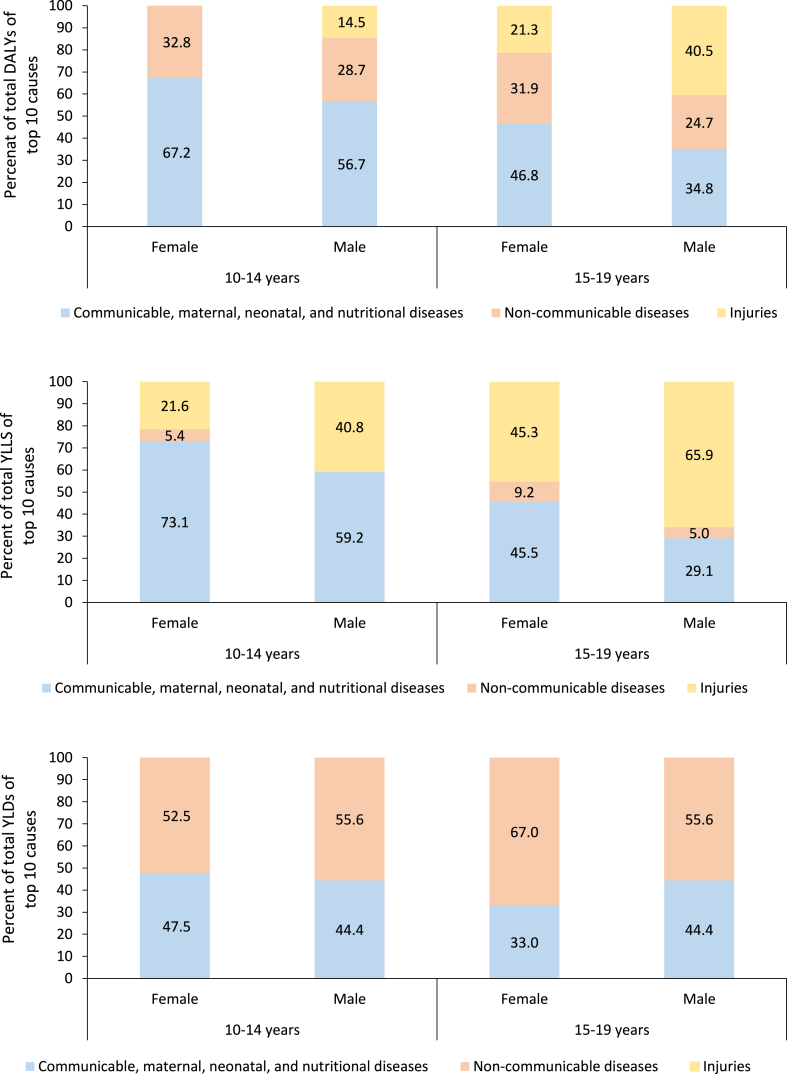
**Percentage contribution of major disease groups to the total disability-adjusted life years (DALYs), years of life lost (YLLs), and years lived with disability (YLDs) of top 10 causes among adolescent aged 10–14 years and 15–19 years by sex in India in 2019, Global Burden of Disease Study**.

An estimated 6.75 million and 9.25 million DALYs were attributed to females in the 10–14 years and 15–19 years age groups, 39.1% and 44.2% of it accounted for by YLLs, respectively. The distribution of the top 10 causes of DALYs, YLLs and YLDs for females by age group is shown in Fig. 1 and Appendix p 3. In the 10–14 years, five out of the top 10 causes of DALYs were CMNNDs contributing 67.2% of the total DALYs and the remaining were NCDs contributing 32.8% of the total DALYs. This pattern changed when considering only the leading 10 causes of YLLs, wherein six of the 10 leading causes of YLLs were CMNNDs contributing 73.1% of the total YLLs, three were injuries contributing 21.6%, and one was NCD contributing 5.4%. Seven out of the top 10 causes of YLDs in this age group were NCDs contributing 52.5% of the total YLDs and the remaining burden was from CMNNDs.

The pattern in the top 10 causes of DALYs and YLLs in 15–19 years was different than the 10–14 years age group, as injuries contributed to 21.3% and 45.3% to the total respective DALYs and YLLs (Fig. 1 and Appendix p 3). Eight out of the top 10 causes of YLDs were from NCDs contributing 67.0% and the remaining burden was from CMNNDs (Fig. 1 and Appendix p 3).

### Disease burden among male adolescents

An estimated 6.71 million and 9.65 million DALYs were attributed to males in 10–14 years and 15–19 years age groups, 42.3% and 41.1% of it accounted for by YLLs, respectively. The distribution of the top 10 causes of DALYs, YLLs and YLDs for males by age group is shown in Fig. 1 and Appendix p 4. In 10–14 years, four out of the leading 10 causes of DALYs were CMNNDs contributing 56.7% of the total DALYs from these diseases, four were NCDs contributing 28.7%, and two were injuries contributing 14.5%. On considering only the leading 10 causes of YLLs in this age group, half of the top 10 causes of YLLs were CMNNDs accounting for 59.2% of the total YLLs from these causes and the remaining half were from injuries contributing 40.8%. Seven out of the top 10 causes of YLDs in this age group were from NCDs contributing 55.6% of the total YLDs from these top causes, and the remaining burden was from CMNNDs.

The pattern in the top 10 causes of DALYs and YLLs among males aged 15–19 years was different than the 10–14 years age group, as injuries contributed to 40.5% and 65.9% of the total respective DALYs and YLLs (Fig. 1 and Appendix p 4). Eight out of the top 10 causes of YLDs for 15–19 years were from NCDs contributing 55.6% and the remaining burden was from CMNNDs (Fig. 1 and Appendix p 4).

### Comparison of disease burden among female and male adolescents

The majority of the leading 10 causes of DALYs among adolescents aged 10–14 years were similar for females and males, except that lower respiratory infections and low back pain were seen only for females whereas road injuries and drowning were seen only for males (Appendix pp 3 and 4). Dietary iron deficiency was the leading cause of YLDs in both females and males aged 10–14 years, followed by headache disorder (8.9%, 95% UI 0.5–19.4) and neonatal disorders (5.4%, 95% UI 3.8–7.4) for females, and conduct disorders (8.8%, 95% UI 5.6–13.1) and neonatal disorders for males (7.3%, 95% UI 5.1–9.8) as shown in Appendix pp 3 and 4. The DALYs and YLLs of drowning and road traffic injuries were statistically significantly higher for males than females, and the burden of other top 10 leading diseases/conditions was similar between males and females in this age group.

Among the 15–19 years, self-harm was among the top two leading causes of disease burden for both females and males in terms of DALYs and YLLs; road injuries were the leading cause of DALYs and YLLs in males (Appendix pp 3 and 4). The leading 10 causes of YLDs were similar for females and males, except that, gynaecological disorders and hemoglobinopathies and hemolytic anemias were among the leading causes only for females, and conduct disorder and age-related hearing loss were among the leading causes only for males (Appendix pp 3 and 4). The DALYs, YLLs and YLDs for dietary iron deficiency, self-harm, fire, heat, and hot substances were statistically significantly higher for females than males; and that for road traffic injuries, drowning, interpersonal violence and conduct disorder were statistically significantly higher for males than for females in this age group.

### Variation in disease burden across states

There was substantial variation in the top 10 causes of DALYs across the states of India by age group and sex in 2019 (appendix pp 5–8). Several of the causes of DALYs that were not in the top 10 list for India could be seen for the states. For instance, malaria was among the leading cause of DALYs in Odisha, Chhattisgarh, Jharkhand, Tripura, and Goa among females aged 10–14 years but was not on the list of leading causes for India (Appendix p 5). For females in this age group, injuries such as drowning, self-harm, road injuries, and animal contact could be seen in the list of top 10 causes across the states, but not for India (Appendix p 5). Anxiety disorder was seen on the list of top 10 causes for several of the less and more developed states among females aged 10–14 years and 15–19 years but not among the top 10 causes for India in these age groups (Appendix pp 5 and 6).

Malaria was the leading cause of DALYs also in males aged 10–14 years in the states of Odisha, Chhattisgarh and Jharkhand (Appendix p 7). Asthma, dermatitis, idiopathic developmental intellectual disability, idiopathic epilepsy, animal contact, and falls were on the list of top 10 causes of DALYs in several states for males aged 10–14 years but not in the list of leading causes for India in this age group (Appendix p 7). For males aged 15–19 years, causes such as tuberculosis, idiopathic epilepsy, acne vulgaris, falls, and interpersonal violence were on the list of leading causes across several states but these were not among the leading causes in India for this age group (Appendix p 8).

### Review of IAHS and its indicators

Among the leading 10 causes of YLLs for adolescent females and males in the 10–14 years and 15–19 years in India, only maternal disorders, self-harm, and ischemic heart diseases are explicitly covered under the 6 IAHS themes in the 15–19 years females, and self-harm in this age group for males (Table 1). Among the DALYs, dietary iron deficiency in both females and males in the 10–14 years and females 15–19 years age groups, maternal disorders and self-harm in females 15–19 years old and depressive disorders and self-harm in males 15–19 years old are explicitly covered under the 6 IAHS themes (Table 2). It is important to note that the theme of preventing injuries and violence or the specific strategies proposed under this theme in the IAHS do not explicitly mention any specific injury other than gender-based violence (Table 1, Table 2). The IAHS operational framework mentions the provision of counselling for sexual assault, rape, domestic violence, road traffic accidents, and drowning under the AFMC service package.

**Table 1:** Coverage of the 10 leading causes of years of life lost (YLLs) among adolescents in India in 2019 as per the Global Burden of Disease Study under the India Adolescent Health Strategy (IAHS).

∗Suicidal tendencies are stated under the mental health theme (Panel 1).

†Road injuries, falls, interpersonal violence, drowning, animal contact, fire, heat and hot substances, and other unintentional injuries are not explicitly listed under the preventing injuries and violence theme (Panel 1).

**Table 2:** Coverage of the 10 leading causes of disability-adjusted life years (DALYs) among adolescents in India in 2019 as per the Global Burden of Disease Study under the India Adolescent Health Strategy (IAHS).

∗Suicidal tendencies are stated under the mental health theme (Panel 1).

†Road injuries, falls, drowning, and animal contact are not explicitly listed under the preventing injuries and violence theme (Panel 1).

^‡^Gynaecological diseases are not explicitly stated under the sexual and reproductive health theme (Panel 1).

The availability and age- and sex-disaggregation of service delivery indicators in AFHC MIS for adolescents is shown in Appendix p 9. The AFHC MIS covers the service delivery indicators under the 6 IAHS themes, however, NCDs covered in the AFHC MIS are different (skin problems) than those listed in the IAHS (hypertension, stroke, cardiovascular diseases, and diabetes). Overall, the service delivery indicators were captured for the two age groups disaggregated by sex, except those that are applicable for females in AFHC MIS. The availability and age- and sex-disaggregation of adolescent health or service delivery indicators available in HMIS are shown in Appendix p 10. HMIS includes only a few service delivery indicators for adolescents covering 2 of the 6 IAHS themes namely, nutrition and reproductive health, with the latter focussed on females and neither disaggregated by age groups. The capture of deaths by specific cause of death is listed for a variety of CMNNDs, NCDs, and injuries but is combined for adolescents and adults, and there is no sex disaggregation.

The availability and age- and sex-disaggregation of the IAHS outcome indicators from population-level data sources is shown in Table 3. The data capture for outcome indicators for adolescents aged 10–14 years irrespective of sex was largely missing from these sources. For adolescents aged 15–19 years, data were captured on many of the IAHS indicators for both females and males, except that the sexual and reproductive health and sexual and physical violence indicators were captured only for females. None of the surveys captured data for either females or males aged 15–19 years for physical inactivity and mental health indicators.

**Table 3:** Availability of outcome indicators for monitoring of the India Adolescent Health Strategy (IAHS) at the population-level, disaggregated by age and gender.

## Discussion

The IAHS provides a strategy framework for the country to operationalise the services needed to address the health needs of adolescents. Considerable differences are seen in the thematic focus of the IAHS as compared with the top ten leading causes of fatal and non-fatal disease burden in adolescents in India, and significant gaps are seen in the availability of population-level data to monitor the outcome indicators of the IAHS. The findings of this paper provide feedback on areas in which improvements can be made to promote the health of adolescents in India.

Within the 6 thematic areas of the IAHS, the most strategies and indicators identified are for sexual and reproductive health followed by nutrition, and broadly these account for YLDs and not YLLs.^4^ Significant gaps in comparison to the disease burden are seen across injuries, communicable diseases, and NCDs which can have serious implications for improving adolescent health in India. Injuries accounted for 66% and 45% of YLLs in males and females aged 15–19 years, and 41% in males aged 10–14 years. Specifically, road injuries and self-harm accounted for most of the injury deaths in the 15–19 years whereas drowning and road injuries accounted for the most injury deaths in the 10–14 years males. The IAHS uses the term “injuries, sexual abuse, domestic violence and gender-based violence” under the theme of injuries/violence,^4^ and the IAHS operational framework in addition mentions road traffic accidents and drowning.^6^ Screening for suicidal tendencies is indicated as a strategy under the mental health theme, and management of it is linked to the existing mental health services.^4^ The burden of road injuries and self-harm in this age group and its gendered nature, and the economic and societal implications of loss of young lives have been reported previously.^29^^,^^30^ Drowning-related morbidity and mortality also have been reported to have a significant impact in India.^31^ Importantly, the risk factors and interventions for the variety of injuries are different and generic injury strategies proposed in the IAHS cannot address these injuries effectively.^32^, ^33^, ^34^, ^35^, ^36^ As indicated by this analysis, further specification of strategies, interventions and indicators by the type of injury is urgently needed in the IAHS to address the burden of injury-related deaths in adolescents in India.

Diarrhoea, lower respiratory infections, malaria, encephalitis, tuberculosis, typhoid, cirrhosis, and hepatitis are the other disease conditions accounting for YLLs and DALYs in adolescents but these are not covered under the IAHS and service provision for these is also not specifically tracked under the AFHC MIS.^4^^,^^16^ Furthermore, the cause of death data captured in the HMIS does not allow for an understanding of the mortality burden by either age or sex, thereby, restricting the usefulness of these data for surveillance and planning. It may be likely that the various disease-specific national health programs capture mortality data within the specific programs, which may be available by age and sex, however, these data are not available in the public domain.^37^

This analysis has highlighted both the sex differentials and similarities in the disease burden in both age groups. It has to be noted that the similarities in the top ten disease/conditions between the two sexes do not necessarily mean that the magnitude of the particular disease/condition is the same for both sexes. Furthermore, recent research has highlighted how in early to middle adolescence, both conformity and non-conformity to gender-typed behaviours simultaneously affect behaviours that are risk-increasing or protective.^38^ On one hand masculinity promotes healthy physical activity, and on the other, it can also encourage unhealthy diets, tobacco use, and alcohol consumption, which are behaviours that contribute to non-communicable disease burden in the long term. Similarly, though femininity decreases the risk of substance abuse, it increases greater susceptibility to depression contributing to the gender divide in mental health disorders that persist over the life course.^38^^,^^39^ Gender equity is among the guiding principles of the IAHS,^4^ and it emphasises addressing the needs of adolescents of different genders in an equitable, non-discriminatory manner because deep-rooted gender stereotyping and differentials result in health risks, and has also specified needs-based programme planning with gender equity as a central theme to be ensured. However, neither the IAHS nor the IAHS operational framework has explicitly identified the gendered pathways or gender-transformative interventions that challenge gender ideologies to address gender inequities in early adolescence before identities solidify, because gender intensification seems to follow as a social extension of adolescence, peaking in early adulthood.^39^, ^40^, ^41^

A recent review of evidence on the health determinants of criminalisation among adolescents has highlighted that several health and development conditions in adolescence such as neurodevelopmental disability, poor mental health, trauma, and experiences of maltreatment can increase the risk that a young person will be exposed to the criminal justice system.^42^ The analysis presented in this paper indicates that idiopathic developmental intellectual disability, interpersonal violence, conduct disorder, and depressive disorders are among the top 10 diseases/conditions of DALYs among adolescents in India. The identification of marginalised and vulnerable adolescents such as out-of-school adolescents, those living in remote areas, very young adolescents, and those with physical or mental disabilities and developing specific communication strategies to reach them are strategic imperatives in the IAHS.^4^ The evidence on the health determinants of criminalisation among adolescents and the disease burden findings in this paper provide a compelling case for investment by the IAHS in approaches to prevent criminalisation in adolescents related to health and developmental difficulties and to better address related needs for those within a criminal justice system.^42^

The 10–14 years age group was noticeably missing across most themes, and the 15–19 years age group was missing for physical inactivity and mental health themes for both sexes from the population-based data sources suggested to monitor the IAHS outcome indicators. The World Health Organization has recommended the collection of high-quality, internationally comparable data as essential to support international policy development and monitor progress towards global targets. For example, the Health Behaviour in School-aged Children study undertaken in 45 countries across Europe and North America, has been used to underpin not only the WHO European strategy for child and adolescent health providing a road map for countries and regions to engage across sectors to promote the health and well-being of children and adolescents, but also for monitoring progress on their health priorities and compare with other similar countries and regions.^43^ Furthermore, the varied pattern of disease burden among adolescents at the state level in India will require nationally comparable data to support state policy development and monitor the progress of adolescent health. The considerable sub-national heterogeneity in the disease burden of the leading causes among adolescents provides crucial insights that can help titrate the response to the specific needs of each state.

Comparing the top 10 leading causes of YLLs and YLDs for adolescents in India in 2019 with that in 2014 when the IAHS was launched,^44^ highlighted that tuberculosis, acute hepatitis, and other transport injuries were in the top 10 in 2014 and are not so in 2019. Encephalitis, falls, and congenital birth defects are in the top 10 both in 2019 and 2014. However, none of these diseases/conditions were specifically addressed in the IAHS. In light of the findings presented in this paper, it may be useful for IAHS to consider appropriate revisions to address priority health themes and to implement a mechanism to collect population-level outcome indicators to monitor child and adolescent health in a comparable manner across India to strengthen the impact of IAHS. A set of priority health indicators for adolescent health measurement are also proposed by the Global Action for Measurement of Adolescent Health Advisory Group,^45^ which may need consideration for inclusion in IAHS as well. Investment is also needed in longer periods of observation to fully evaluate the contribution of gender to individual health trajectories and the overall burden of disease, and to identify and quantify the gendered pathways contributing to health disparities, including differences in exposures and vulnerabilities, behaviours, and health-care use and response.^46^ UDAYA, a longitudinal study has established the levels, patterns and trends in the disease/conditions for 10–19 years old in two Indian states, and has provided robust insights on how and where to make investments in adolescents to influence their life course by the time they reach young adulthood and beyond.^47^

There are some limitations to the analysis presented. The IAHS review undertaken within the context of disease burden is based on the review of strategy and operational framework documentation available in the public domain, and not based on the actual implementation of the program on the ground such as the availability and utilisation of services under the IAHS as it was beyond the scope of the analysis undertaken.^48^, ^49^, ^50^, ^51^, ^52^ Also, the assessment of quality and completeness of the AFHC MIS and HMIS were beyond the scope of this study. The general limitations of GBD methods are published elsewhere.^12^, ^13^, ^14^, ^15^^,^^53^ A specific limitation for India is an incomplete medically certified cause of death system that covers only a small proportion of the deaths in India and has variable coverage across the states.^54^ Additionally, the COVID-19 pandemic has caused huge social and economic disruption to young people, with unprecedented interruptions in education, and profound effects on mental health, and the consequences in many countries will probably be reversals in hard-fought gains in adolescent sexual and reproductive health, food security and nutrition, education, and employment,^55^ which are beyond the scope of this paper. The strengths of the analysis presented are the comparable disease burden as estimated by the GBD for the two age groups, by sex and by state, and the availability of this disease burden considering fatal and non-fatal health outcomes that allowed for more in-depth interpretation of the disease burden focus within the IAHS. The review of IAHS, AFHC MIS and HMIS within the context of disease burden, and the gaps highlighted in the availability of outcome indicators are major strengths of the analysis presented.

In conclusion, the IAHS provides a template for India to ensure that all adolescents in India are able to realize their full potential by making informed and responsible decisions related to their health and well-being. The findings in this paper provide evidence to the decision makers for re-organising the health themes and strategies in the IAHS based on the disease burden trends in the country for adolescents by age, sex and state for improved adolescent health and wellbeing.

## Contributors

RD, GAK and LD conceptualised the study. RD drafted the manuscript. AP performed the data analysis with contributions from RD and GAK. All authors contributed to the interpretation and agreed with the final version of the paper. GAK and AP verified the data underlying this study. All authors had full access to all the data in the study and had the final responsibility for the decision to submit for publication.

## Data sharing statement

The data used for estimating disease burden in this paper are available at http://ghdx.healthdata.org/gbd-2019, https://vizhub.healthdata.org/gbd-compare/india, and from the authors on request.

## Declaration of interests

The authors declare that they have no competing interests.
