# Corrigendum to “Review of the India Adolescent Health Strategy in the context of disease burden among adolescentstitle of article” [The Lancet Regional Health – Southeast Asia 20 (2024) 100283]

**DOI:** 10.1016/j.lansea.2024.100397

**Published:** 2024-04-04

**Authors:** Rakhi Dandona, Anamika Pandey, G Anil Kumar, Monika Arora, Lalit Dandona

**Affiliations:** a Public Health Foundation of India, New Delhi, India; b Institute for Health Metrics and Evaluation, University of Washington, Seattle, USA

The authors regret that there are a few errors in the published version of this paper.

1. In graph 3 of Fig. 1, the percentage of NCDs to the total YLDs for males 15–19 years should be 75.8% and that of CMNNDs should be 24.2%. In the y-axis title for graph 1 in Fig. 1 “percenat” should correct to “percent” and for graph 2 in Fig. 1 “YLLS” should correct to “YLLs”. The correct figure is provided below. To reflect the edits in graph 3 of Fig. 1, the percentage contribution of NCDs to total YLDs reported in the results section on page 4 paragraph 4 last line as “55.6%” should correct to “75.8%”.

**Fig. 1:**
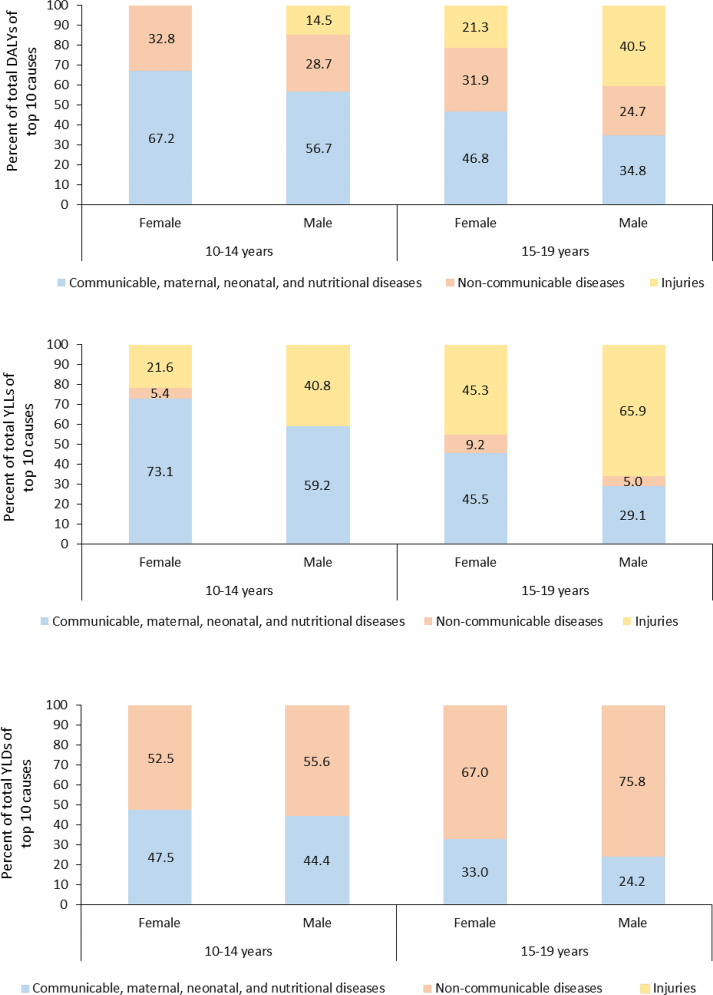
**Percentage contribution of major disease groups to the total disability-adjusted life years (DALYs), years of life lost (YLLs), and years lived with disability (YLDs) of top 10 causes among adolescent aged 10–14 years and 15–19 years by sex in India in 2019, Global Burden of Disease Study**.
2. Reference 44 citation should correct to “Institute for Health Metrics and Evaluation (IHME). GBD Compare. Seattle, WA: IHME, University of Washington, 2019. https://vizhub.healthdata.org/gbd-compare/. Accessed March 27, 2023.”
3. On page 3 paragraph 1 “Institute of Health Metrics and Evaluation's” should correct to “Institute for Health Metrics and Evaluation's”.

The authors would like to apologise for any inconvenience caused.

