# Supplementary material for: Review of the India Adolescent Health Strategy in the context of disease burden among adolescents
Source: Lancet Reg Health Southeast Asia. 2023 Sep 28;20:100283. doi: 10.1016/j.lansea.2023.100283 (PMC10794100; doi:10.1016/j.lansea.2023.100283)
Supplement: Supplementary Tables 1–6 [file mmc1.docx]

**Review of the India Adolescent Health Strategy**

**in the context of disease burden among adolescents**

**Web Appendix**

Correspondence to: Prof. Rakhi Dandona, [rakhi.dandona@phfi.org](mailto:rakhi.dandona@phfi.org)

**Table of contents**

[Table 1. Top 10 causes of disability-adjusted life years (DALYs), years of life lost (YLLs), and years lived with disability (YLDs) among female adolescents aged 10-14 years and 15-19 years in India in 2019, the Global Burden of Disease Study. 3](#_Toc131803940)

[Table 2. Top 10 causes of disability-adjusted life years (DALYs), years of life lost (YLLs), and years lived with disability (YLDs) among male adolescents aged 10-14 years and 15-19 years in India in 2019, the Global Burden of Disease Study. 4](#_Toc131803941)

[Table 3. Top 10 causes of disability-adjusted life years (DALYs) among adolescents aged 10-14 years and 15-19 years for females in the states of India in 2019, the Global Burden of Disease Study. 5](#_Toc131803942)

[Table 4. Top 10 causes of disability-adjusted life years (DALYs) among adolescents aged 10-14 years and 15-19 years for males in the states of India in 2019, the Global Burden of Disease Study. 7](#_Toc131803943)

[Table 5. Adolescent health indicators captured in the Adolescent Friendly Health Clinic Management Information System (AFHC MIS). 9](#_Toc131803944)

[Table 6. Adolescent health indicators available in the Health Management Information System (HMIS). 10](#_Toc131803945)

# **Table 1.** **Top 10 causes of disability-adjusted life years (DALYs), years of life lost (YLLs), and years lived with disability (YLDs) among female adolescents aged 10-14 years and 15-19 years in India in 2019, the Global Burden of Disease Study.**

| **10-14 years** | | | | | | **15-19 years** | | | | | |
| --- | --- | --- | --- | --- | --- | --- | --- | --- | --- | --- | --- |
| **DALYs** | | **YLLs** | | **YLDs** | | **DALYs** | | **YLLs** | | **YLDs** | |
| **Top 10 individual causes** | **Percent of total DALYs (95% UIs)** | **Top 10 individual causes** | **Percent of total YLLs (95% UIs)** | **Top 10 individual causes** | **Percent of total YLDs (95% UIs)** | **Top 10 individual causes** | **Percent of total DALYs (95% UIs)** | **Top 10 individual causes** | **Percent of total YLLs (95% UIs)** | **Top 10 individual causes** | **Percent of total YLDs (95% UIs)** |
| Dietary iron deficiency | 11.4 (8.6-14.1) | Typhoid and paratyphoid | 18.5 (8.3-33.3) | Dietary iron deficiency | 18.8 (15.2-22.2) | Self-harm | 9.7 (7.7-12.1) | Self-harm | 21.7 (18.8-24.4) | Dietary iron deficiency | 15.0 (12.0-18.0) |
| Typhoid and paratyphoid | 7.4 (3.2-13.5) | Diarrheal diseases | 10.5 (3.6-21.1) | Headache disorders | 8.9 (0.5-19.4) | Dietary iron deficiency | 8.3 (6.2-10.6) | Typhoid and paratyphoid | 7.5 (2.7-14.9) | Headache disorders | 10.5 (1.2-21.6) |
| Diarrheal diseases | 6.3 (3.4-10.7) | Lower respiratory infections | 5.3 (3.9-6.8) | Neonatal disorders | 5.4 (3.8-7.4) | Headache disorders | 5.9 (0.6-12.9) | Maternal disorders | 7.1 (5.7-8.6) | Depressive disorders | 5.6 (3.9-7.8) |
| Headache disorders | 5.4 (0.3-12.5) | Animal contact | 5.0 (2.4-7.7) | Conduct disorder | 4.5 (2.6-7.2) | Diarrheal diseases | 4.0 (2.1-7.4) | Diarrheal diseases | 6.3 (2.1-13.8) | Gynecological diseases | 5.1 (3.2-7.7) |
| Neonatal disorders | 3.2 (2.5-4.2) | Self-harm | 4.7 (3.6-5.7) | Hemoglobinopathies and hemolytic anemias | 3.6 (2.9-4.3) | Typhoid and paratyphoid | 3.4 (1.2-6.8) | Tuberculosis | 4.1 (3.2-5.1) | Low back pain | 4.5 (2.9-6.8) |
| Conduct disorder | 2.7 (1.5-4.4) | Malaria | 4.6 (1.7-10.3) | Diarrheal diseases | 3.6 (2.5-4.8) | Maternal disorders | 3.3 (2.4-4.3) | Cirrhosis and other chronic liver diseases | 3.1 (2.5-3.9) | Neonatal disorders | 4.1 (2.9-5.5) |
| Hemoglobinopathies and hemolytic anemias | 2.4 (1.9-2.9) | Drowning | 3.8 (2.9-4.7) | Idiopathic developmental intellectual disability | 3.4 (2.2-4.8) | Depressive disorders | 3.1 (2.2-4.4) | Road injuries | 3.0 (2.5-3.6) | Anxiety disorders | 3.9 (2.7-5.5) |
| Lower respiratory infections | 2.2 (1.5-2.9) | Encephalitis | 3.6 (2.3-6.7) | Anxiety disorders | 3.4 (2.2-5.0) | Gynaecological diseases | 2.9 (1.8-4.5) | Fire, heat, and hot substances | 2.7 (1.3-4.8) | Acne vulgaris | 3.3 (2.1-4.7) |
| Idiopathic developmental intellectual disability | 2.1 (1.3-3.0) | Congenital birth defects | 3.3 (2.4-4.5) | Low back pain | 3.4 (2.2-5.1) | Low back pain | 2.5 (1.6-3.7) | Lower respiratory infections | 2.5 (2.1-3.2) | Hemoglobinopathies and hemolytic anemias | 3.0 (2.4-3.6) |
| Low back pain | 2.1 (1.3-3.1) | Acute hepatitis | 3.0 (2.0-5.1) | Dermatitis | 3.0 (1.9-4.7) | Tuberculosis | 2.3 (1.8-2.9) | Ischemic heart disease | 2.5 (2.1-2.8) | Idiopathic developmental intellectual disability | 2.6 (1.6-3.6) |
|  |  |  |  |  |  |  |  |  |  |  |  |

UIs=Uncertainty intervals

|  | Communicable, maternal, neonatal, and nutritional diseases |
| --- | --- |
|  | Non-communicable disease |
|  | Injuries |

# **Table 2. Top 10 causes of disability-adjusted life years (DALYs), years of life lost (YLLs), and years lived with disability (YLDs) among male adolescents aged 10-14 years and 15-19 years in India in 2019, the Global Burden of Disease Study.**

| **10-14 years** | | | | | | **15-19 years** | | | | | |
| --- | --- | --- | --- | --- | --- | --- | --- | --- | --- | --- | --- |
| **DALYs** | | **YLLs** | | **YLDs** | | **DALYs** | | **YLLs** | | **YLDs** | |
| **Top 10 individual causes** | **Percent of total DALYs (95% UIs)** | **Top 10 individual causes** | **Percent of total YLLs (95% UIs)** | **Top 10 individual causes** | **Percent of total YLDs (95% UIs)** | **Top 10 individual causes** | **Percent of total DALYs (95% UIs)** | **Top 10 individual causes** | **Percent of total YLLs (95% UIs)** | **Top 10 individual causes** | **Percent of total YLDs (95% UIs)** |
| Typhoid and paratyphoid | 8.8 (3.8-15.9) | Typhoid and paratyphoid | 20.3 (9.2-36.2) | Dietary iron deficiency | 13.7 (10.9-16.5) | Road injuries | 8.6 (6.4-10.6) | Road injuries | 15.3 (11.0-18.0) | Headache disorders | 9.3 (1.1-19.1) |
| Dietary iron deficiency | 7.8 (5.8-10.0) | Drowning | 7.7 (5.8-9.6) | Conduct disorder | 8.8 (5.6-13.1) | Self-harm | 5.6 (4.1-7.4) | Self-harm | 11.3 (8.7-14.2) | Neonatal disorders | 6.6 (4.7-8.9) |
| Diarrheal diseases | 5.2 (3.5-9.3) | Road injuries | 6.9 (4.7-8.6) | Neonatal disorders | 7.3 (5.1-9.8) | Typhoid and paratyphoid | 4.8 (1.7-9.2) | Typhoid and paratyphoid | 9.4 (3.4-18.2) | Conduct disorder | 5.4 (3.3-8.1) |
| Conduct disorder | 5.1 (3.1-7.6) | Diarrheal diseases | 6.6 (3.4-15.7) | Headache disorders | 6.2 (0.4-13.2) | Headache disorders | 4.7 (0.5-10.2) | Drowning | 4.8 (3.9-6.0) | Dietary iron deficiency | 5.4 (3.9-7.2) |
| Neonatal disorders | 4.1 (3.1-5.3) | Animal contact | 4.9 (3.0-6.8) | Diarrheal diseases | 4.1 (2.9-5.6) | Diarrheal diseases | 3.5 (2.4-6.5) | Diarrheal diseases | 4.3 (2.1-10.4) | Depressive disorders | 5.2 (3.6-7.2) |
| Headache disorders | 3.6 (0.2-7.9) | Malaria | 4.1 (1.6-9.2) | Idiopathic developmental intellectual disability | 3.8 (2.4-5.3) | Neonatal disorders | 3.3 (2.5-4.3) | Tuberculosis | 3.9 (3.0-4.8) | Acne vulgaris | 3.8 (2.4-5.5) |
| Road injuries | 3.4 (2.4-4.3) | Lower respiratory infections | 3.7 (2.8-4.6) | Dermatitis | 3.5 (2.2-5.4) | Conduct disorder | 2.7 (1.6-4.1) | Interpersonal violence | 3.5 (2.7-4.6) | Anxiety disorders | 3.4 (2.4-4.9) |
| Drowning | 3.3 (2.4-4.5) | Falls | 3.4 (2.4-4.5) | Hemoglobinopathies and hemolytic anemias | 3.4 (2.7-4.1) | Dietary iron deficiency | 2.7 (1.8-3.8) | Cirrhosis and other chronic liver diseases | 3.0 (2.3-3.9) | Idiopathic developmental intellectual disability | 3.4 (2.1-4.8) |
| Idiopathic developmental intellectual disability | 2.2 (1.3-3.2) | Self-harm | 3.3 (2.2-4.6) | Age-related and other hearing loss | 3.1 (2.1-4.5) | Depressive disorders | 2.6 (1.8-3.7) | Other unintentional injuries | 2.5 (0.6-3.5) | Age-related and other hearing loss | 3.4 (2.3-4.6) |
| Hemoglobinopathies and hemolytic anemias | 2.2 (1.7-2.7) | Encephalitis | 3.2 (2.1-6.3) | Anxiety disorders | 2.3 (1.5-3.5) | Drowning | 2.4 (1.8-3.1) | Falls | 2.5 (1.9-3.3) | Low back pain | 3.2 (2.0-4.8) |

UIs=Uncertainty intervals

|  | Communicable, maternal, neonatal, and nutritional diseases |
| --- | --- |
|  | Non-communicable disease |
|  | Injuries |

# **Table 3.** **Top 10 causes of disability-adjusted life years (DALYs) among adolescents aged 10-14 years and 15-19 years for females in the states of India in 2019, the Global Burden of Disease Study.**

**a) 10-14 years**

| **Cause of DALYs** | **Rank 1** | **Rank 2** | **Rank 3** | **Rank 4** | **Rank 5** | **Rank 6** | **Rank 7** | **Rank 8** | **Rank 9** | **Rank 10** |
| --- | --- | --- | --- | --- | --- | --- | --- | --- | --- | --- |
| **India** | **Dietary iron deficiency** | **Typhoid and paratyphoid** | **Diarrheal diseases** | **Headache disorders** | **Neonatal disorders** | **Conduct disorder** | **Hemoglobinopathies and hemolytic anemias** | **Lower respiratory infections** | **Idiopathic developmental intellectual disability** | **Low back pain** |
| **Less developed states** | | | | | | | | | | |
| Assam | Dietary iron deficiency | Typhoid and paratyphoid | Diarrheal diseases | Headache disorders | Neonatal disorders | Drowning | Acute hepatitis | Conduct disorder | Lower respiratory infections | Hemoglobinopathies and hemolytic anemias |
| Bihar | Dietary iron deficiency | Diarrheal diseases | Headache disorders | Typhoid and paratyphoid | Neonatal disorders | Idiopathic developmental intellectual disability | Conduct disorder | Hemoglobinopathies and hemolytic anemias | Animal contact | Encephalitis |
| Chhattisgarh | Dietary iron deficiency | Malaria | Typhoid and paratyphoid | Diarrheal diseases | Headache disorders | Neonatal disorders | Conduct disorder | Hemoglobinopathies and hemolytic anemias | Animal contact | Low back pain |
| Jharkhand | Dietary iron deficiency | Malaria | Headache disorders | Diarrheal diseases | Typhoid and paratyphoid | Neonatal disorders | Conduct disorder | Hemoglobinopathies and hemolytic anemias | Idiopathic developmental intellectual disability | Anxiety disorders |
| Madhya Pradesh | Dietary iron deficiency | Typhoid and paratyphoid | Diarrheal diseases | Headache disorders | Neonatal disorders | Hemoglobinopathies and hemolytic anemias | Conduct disorder | Idiopathic developmental intellectual disability | Low back pain | Animal contact |
| Odisha | Malaria | Dietary iron deficiency | Diarrheal diseases | Typhoid and paratyphoid | Headache disorders | Neonatal disorders | Conduct disorder | Idiopathic epilepsy | Animal contact | Hemoglobinopathies and hemolytic anemias |
| Rajasthan | Typhoid and paratyphoid | Dietary iron deficiency | Diarrheal diseases | Headache disorders | Lower respiratory infections | Neonatal disorders | Animal contact | Conduct disorder | Hemoglobinopathies and hemolytic anemias | Drowning |
| Uttar Pradesh | Dietary iron deficiency | Typhoid and paratyphoid | Diarrheal diseases | Headache disorders | Lower respiratory infections | Animal contact | Neonatal disorders | Conduct disorder | Hemoglobinopathies and hemolytic anemias | Idiopathic developmental intellectual disability |
| Uttarakhand | Dietary iron deficiency | Typhoid and paratyphoid | Headache disorders | Neonatal disorders | Diarrheal diseases | Conduct disorder | Hemoglobinopathies and hemolytic anemias | Low back pain | Anxiety disorders | Lower respiratory infections |
| **More developed states** | | | | | | | | | | |
| Andhra Pradesh | Dietary iron deficiency | Headache disorders | Typhoid and paratyphoid | Diarrheal diseases | Neonatal disorders | Conduct disorder | Hemoglobinopathies and hemolytic anemias | Malaria | Low back pain | Anxiety disorders |
| Arunachal Pradesh | Dietary iron deficiency | Headache disorders | Typhoid and paratyphoid | Neonatal disorders | Diarrheal diseases | Conduct disorder | Low back pain | Anxiety disorders | Dermatitis | Idiopathic developmental intellectual disability |
| Delhi | Dietary iron deficiency | Typhoid and paratyphoid | Headache disorders | Neonatal disorders | Diarrheal diseases | Conduct disorder | Hemoglobinopathies and hemolytic anemias | Anxiety disorders | Dermatitis | Low back pain |
| Goa | Headache disorders | Malaria | Neonatal disorders | Dietary iron deficiency | Conduct disorder | Diarrheal diseases | Low back pain | Dermatitis | Congenital birth defects | Depressive disorders |
| Gujarat | Dietary iron deficiency | Typhoid and paratyphoid | Headache disorders | Diarrheal diseases | Neonatal disorders | Self-harm | Conduct disorder | Lower respiratory infections | Animal contact | Malaria |
| Haryana | Dietary iron deficiency | Typhoid and paratyphoid | Headache disorders | Diarrheal diseases | Neonatal disorders | Conduct disorder | Hemoglobinopathies and hemolytic anemias | Road injuries | Congenital birth defects | Lower respiratory infections |
| Himachal Pradesh | Dietary iron deficiency | Headache disorders | Typhoid and paratyphoid | Neonatal disorders | Diarrheal diseases | Conduct disorder | Low back pain | Anxiety disorders | Dermatitis | Hemoglobinopathies and hemolytic anemias |
| Jammu and Kashmir & Ladakh | Dietary iron deficiency | Headache disorders | Diarrheal diseases | Neonatal disorders | Conduct disorder | Typhoid and paratyphoid | Congenital birth defects | Low back pain | Anxiety disorders | Lower respiratory infections |
| Karnataka | Dietary iron deficiency | Headache disorders | Diarrheal diseases | Neonatal disorders | Self-harm | Conduct disorder | Typhoid and paratyphoid | Congenital birth defects | Idiopathic epilepsy | Low back pain |
| Kerala | Headache disorders | Dietary iron deficiency | Neonatal disorders | Anxiety disorders | Diarrheal diseases | Conduct disorder | Congenital birth defects | Low back pain | Asthma | Dermatitis |
| Maharashtra | Dietary iron deficiency | Headache disorders | Typhoid and paratyphoid | Diarrheal diseases | Neonatal disorders | Conduct disorder | Low back pain | Anxiety disorders | Dermatitis | Congenital birth defects |
| Manipur | Typhoid and paratyphoid | Headache disorders | Neonatal disorders | Diarrheal diseases | Anxiety disorders | Conduct disorder | Asthma | Low back pain | Idiopathic developmental intellectual disability | Road injuries |
| Meghalaya | Malaria | Dietary iron deficiency | Asthma | Typhoid and paratyphoid | Headache disorders | Neonatal disorders | Diarrheal diseases | Conduct disorder | Low back pain | Idiopathic developmental intellectual disability |
| Mizoram | Typhoid and paratyphoid | Headache disorders | Malaria | Neonatal disorders | Dietary iron deficiency | Diarrheal diseases | Conduct disorder | Low back pain | Anxiety disorders | Dermatitis |
| Nagaland | Typhoid and paratyphoid | Headache disorders | Dietary iron deficiency | Neonatal disorders | Diarrheal diseases | Conduct disorder | HIV/AIDS | Drowning | Low back pain | Anxiety disorders |
| Other small union territories | Dietary iron deficiency | Headache disorders | Neonatal disorders | Conduct disorder | Diarrheal diseases | Typhoid and paratyphoid | Hemoglobinopathies and hemolytic anemias | Anxiety disorders | Low back pain | Self-harm |
| Punjab | Dietary iron deficiency | Typhoid and paratyphoid | Headache disorders | Neonatal disorders | Diarrheal diseases | Conduct disorder | Low back pain | Dermatitis | Congenital birth defects | Anxiety disorders |
| Sikkim | Dietary iron deficiency | Typhoid and paratyphoid | Headache disorders | Neonatal disorders | Diarrheal diseases | Conduct disorder | Low back pain | Anxiety disorders | Lower respiratory infections | Dermatitis |
| Tamil Nadu | Dietary iron deficiency | Self-harm | Headache disorders | Typhoid and paratyphoid | Diarrheal diseases | Neonatal disorders | Congenital birth defects | Conduct disorder | Encephalitis | Drowning |
| Telangana | Dietary iron deficiency | Headache disorders | Typhoid and paratyphoid | Neonatal disorders | Diarrheal diseases | Conduct disorder | Hemoglobinopathies and hemolytic anemias | Low back pain | Anxiety disorders | Dermatitis |
| Tripura | Dietary iron deficiency | Malaria | Headache disorders | Diarrheal diseases | Neonatal disorders | Asthma | Conduct disorder | Typhoid and paratyphoid | Hemoglobinopathies and hemolytic anemias | Anxiety disorders |
| West Bengal | Dietary iron deficiency | Diarrheal diseases | Headache disorders | Self-harm | Drowning | Typhoid and paratyphoid | Neonatal disorders | Conduct disorder | Congenital birth defects | Hemoglobinopathies and hemolytic anemias |

**b) 15-19 years**

| **Cause of DALYs** | **Rank 1** | **Rank 2** | **Rank 3** | **Rank 4** | **Rank 5** | **Rank 6** | **Rank 7** | **Rank 8** | | **Rank 9** | **Rank 10** |
| --- | --- | --- | --- | --- | --- | --- | --- | --- | --- | --- | --- |
| **India** | **Self-harm** | **Dietary iron deficiency** | **Headache disorders** | **Diarrheal diseases** | **Typhoid and paratyphoid** | **Maternal disorders** | **Depressive disorders** | **Gynecological diseases** | | **Low back pain** | **Tuberculosis** |
| **Less developed states** | | | | | | | | | | | |
| Assam | Self-harm | Dietary iron deficiency | Headache disorders | Maternal disorders | Cirrhosis and other chronic liver diseases | Diarrheal diseases | Depressive disorders | Tuberculosis | | Gynaecological diseases | Typhoid and paratyphoid |
| Bihar | Dietary iron deficiency | Headache disorders | Diarrheal diseases | Maternal disorders | Depressive disorders | Gynaecological diseases | Self-harm | Low back pain | | Typhoid and paratyphoid | Hemoglobinopathies and hemolytic anemias |
| Chhattisgarh | Dietary iron deficiency | Headache disorders | Self-harm | Diarrheal diseases | Typhoid and paratyphoid | Gynaecological diseases | Maternal disorders | Low back pain | | Depressive disorders | Tuberculosis |
| Jharkhand | Dietary iron deficiency | Headache disorders | Diarrheal diseases | Self-harm | Depressive disorders | Gynaecological diseases | Low back pain | Maternal disorders | | Neonatal disorders | Anxiety disorders |
| Madhya Pradesh | Dietary iron deficiency | Self-harm | Headache disorders | Typhoid and paratyphoid | Diarrheal diseases | Gynaecological diseases | Depressive disorders | Maternal disorders | | Low back pain | Neonatal disorders |
| Odisha | Self-harm | Dietary iron deficiency | Diarrheal diseases | Headache disorders | Malaria | Typhoid and paratyphoid | Maternal disorders | Depressive disorders | | Idiopathic epilepsy | Tuberculosis |
| Rajasthan | Dietary iron deficiency | Typhoid and paratyphoid | Self-harm | Maternal disorders | Headache disorders | Diarrheal diseases | Tuberculosis | Gynaecological diseases | | Depressive disorders | Road injuries |
| Uttar Pradesh | Dietary iron deficiency | Self-harm | Headache disorders | Diarrheal diseases | Maternal disorders | Typhoid and paratyphoid | Tuberculosis | Gynaecological diseases | | Depressive disorders | Low back pain |
| Uttarakhand | Dietary iron deficiency | Headache disorders | Self-harm | Typhoid and paratyphoid | Depressive disorders | Gynaecological diseases | Neonatal disorders | Low back pain | | Maternal disorders | Diarrheal diseases |
| **More developed states** | | | | | | | | | | | |
| Andhra Pradesh | Dietary iron deficiency | Self-harm | Headache disorders | Depressive disorders | Gynaecological diseases | Low back pain | Diarrheal diseases | Neonatal disorders | | Anxiety disorders | Typhoid and paratyphoid |
| Arunachal Pradesh | Dietary iron deficiency | Headache disorders | Self-harm | Depressive disorders | Gynecological diseases | Low back pain | Neonatal disorders | Cirrhosis and other chronic liver diseases | | Anxiety disorders | Tuberculosis |
| Delhi | Dietary iron deficiency | Headache disorders | Gynaecological diseases | Depressive disorders | Neonatal disorders | Self-harm | Typhoid and paratyphoid | Anxiety disorders | | Low back pain | Acne vulgaris |
| Goa | Headache disorders | Self-harm | Dietary iron deficiency | Depressive disorders | Gynecological diseases | Neonatal disorders | Low back pain | Acne vulgaris | | Anxiety disorders | Diarrheal diseases |
| Gujarat | Self-harm | Dietary iron deficiency | Headache disorders | Tuberculosis | Typhoid and paratyphoid | Diarrheal diseases | Gynecological diseases | Road injuries | | Maternal disorders | Low back pain |
| Haryana | Self-harm | Dietary iron deficiency | Typhoid and paratyphoid | Headache disorders | Depressive disorders | Road injuries | Tuberculosis | Maternal disorders | | Diarrheal diseases | Gynecological diseases |
| Himachal Pradesh | Dietary iron deficiency | Headache disorders | Self-harm | Depressive disorders | Neonatal disorders | Gynecological diseases | Low back pain | Anxiety disorders | | Acne vulgaris | Typhoid and paratyphoid |
| Jammu and Kashmir & Ladakh | Dietary iron deficiency | Headache disorders | Self-harm | Gynecological diseases | Depressive disorders | Low back pain | Neonatal disorders | Anxiety disorders | | Road injuries | Diarrheal diseases |
| Karnataka | Self-harm | Dietary iron deficiency | Headache disorders | Diarrheal diseases | Gynecological diseases | Depressive disorders | Neonatal disorders | Idiopathic epilepsy | | Maternal disorders | Low back pain |
| Kerala | Self-harm | Headache disorders | Dietary iron deficiency | Neonatal disorders | Anxiety disorders | Depressive disorders | Gynecological diseases | Low back pain | | Acne vulgaris | Diarrheal diseases |
| Maharashtra | Self-harm | Dietary iron deficiency | Headache disorders | Depressive disorders | Gynecological diseases | Neonatal disorders | Low back pain | Typhoid and paratyphoid | | Diarrheal diseases | Tuberculosis |
| Manipur | Headache disorders | Depressive disorders | Self-harm | Anxiety disorders | HIV/AIDS | Gynecological diseases | Typhoid and paratyphoid | Low back pain | | Neonatal disorders | Road injuries |
| Meghalaya | Dietary iron deficiency | Headache disorders | Asthma | Typhoid and paratyphoid | Depressive disorders | Gynecological diseases | Maternal disorders | Low back pain | | Neonatal disorders | Diarrheal diseases |
| Mizoram | Headache disorders | Typhoid and paratyphoid | HIV/AIDS | Dietary iron deficiency | Depressive disorders | Gynecological diseases | Low back pain | Neonatal disorders | | Anxiety disorders | Acne vulgaris |
| Nagaland | HIV/AIDS | Headache disorders | Dietary iron deficiency | Depressive disorders | Typhoid and paratyphoid | Gynecological diseases | Neonatal disorders | Low back pain | Anxiety disorders | | Acne vulgaris |
| Other small union territories | Dietary iron deficiency | Self-harm | Headache disorders | Depressive disorders | Neonatal disorders | Gynecological diseases | Low back pain | Anxiety disorders | Acne vulgaris | | Hemoglobinopathies and hemolytic anemias |
| Punjab | Dietary iron deficiency | Headache disorders | Self-harm | Typhoid and paratyphoid | Gynaecological diseases | Depressive disorders | Neonatal disorders | Low back pain | Ischemic heart disease | | Road injuries |
| Sikkim | Dietary iron deficiency | Headache disorders | Self-harm | Depressive disorders | Gynecological diseases | Neonatal disorders | Typhoid and paratyphoid | Low back pain | Anxiety disorders | | Cirrhosis and other chronic liver diseases |
| Tamil Nadu | Self-harm | Dietary iron deficiency | Headache disorders | Depressive disorders | Gynecological diseases | Diarrheal diseases | Low back pain | Neonatal disorders | Road injuries | | Falls |
| Telangana | Dietary iron deficiency | Self-harm | Headache disorders | Depressive disorders | Gynecological diseases | Typhoid and paratyphoid | Diarrheal diseases | Low back pain | Neonatal disorders | | Anxiety disorders |
| Tripura | Self-harm | Dietary iron deficiency | Headache disorders | Depressive disorders | Gynecological diseases | Neonatal disorders | Low back pain | Anxiety disorders | Diarrheal diseases | | Maternal disorders |
| West Bengal | Self-harm | Dietary iron deficiency | Headache disorders | Diarrheal diseases | Depressive disorders | Gynecological diseases | Low back pain | Anxiety disorders | Hemoglobinopathies and hemolytic anemias | | Fire, heat, and hot substances |

# **Table 4. Top 10 causes of disability-adjusted life years (DALYs) among adolescents aged 10-14 years and 15-19 years for males in the states of India in 2019, the Global Burden of Disease Study.**

**a) 10-14 years**

| **Cause of DALYs** | **Rank 1** | **Rank 2** | **Rank 3** | **Rank 4** | **Rank 5** | **Rank 6** | **Rank 7** | **Rank 8** | **Rank 9** | **Rank 10** |
| --- | --- | --- | --- | --- | --- | --- | --- | --- | --- | --- |
| **India** | **Typhoid and paratyphoid** | **Dietary iron deficiency** | **Diarrheal diseases** | **Conduct disorder** | **Neonatal disorders** | **Headache disorders** | **Road injuries** | **Drowning** | **Idiopathic developmental intellectual disability** | **Hemoglobinopathies and hemolytic anemias** |
| **Less developed states** | | | | | | | | | | |
| Assam | Typhoid and paratyphoid | Diarrheal diseases | Dietary iron deficiency | Drowning | Conduct disorder | Neonatal disorders | Headache disorders | Asthma | Road injuries | Acute hepatitis |
| Bihar | Dietary iron deficiency | Diarrheal diseases | Typhoid and paratyphoid | Conduct disorder | Neonatal disorders | Headache disorders | Idiopathic developmental intellectual disability | Hemoglobinopathies and hemolytic anemias | Animal contact | Drowning |
| Chhattisgarh | Malaria | Dietary iron deficiency | Typhoid and paratyphoid | Conduct disorder | Diarrheal diseases | Neonatal disorders | Headache disorders | Drowning | Hemoglobinopathies and hemolytic anemias | Road injuries |
| Jharkhand | Malaria | Dietary iron deficiency | Conduct disorder | Diarrheal diseases | Neonatal disorders | Typhoid and paratyphoid | Headache disorders | Hemoglobinopathies and hemolytic anemias | Idiopathic developmental intellectual disability | Dermatitis |
| Madhya Pradesh | Typhoid and paratyphoid | Dietary iron deficiency | Diarrheal diseases | Drowning | Conduct disorder | Road injuries | Animal contact | Neonatal disorders | Headache disorders | Lower respiratory infections |
| Odisha | Malaria | Typhoid and paratyphoid | Dietary iron deficiency | Diarrheal diseases | Conduct disorder | Asthma | Neonatal disorders | Headache disorders | Drowning | Animal contact |
| Rajasthan | Typhoid and paratyphoid | Dietary iron deficiency | Conduct disorder | Diarrheal diseases | Neonatal disorders | Headache disorders | Road injuries | Drowning | Animal contact | Lower respiratory infections |
| Uttar Pradesh | Typhoid and paratyphoid | Dietary iron deficiency | Diarrheal diseases | Conduct disorder | Road injuries | Neonatal disorders | Headache disorders | Drowning | Animal contact | Idiopathic developmental intellectual disability |
| Uttarakhand | Typhoid and paratyphoid | Dietary iron deficiency | Road injuries | Conduct disorder | Neonatal disorders | Diarrheal diseases | Headache disorders | Drowning | Idiopathic epilepsy | Hemoglobinopathies and hemolytic anemias |
| **More developed states** | | | | | | | | | | |
| Andhra Pradesh | Dietary iron deficiency | Conduct disorder | Neonatal disorders | Headache disorders | Typhoid and paratyphoid | Diarrheal diseases | Asthma | Dermatitis | Hemoglobinopathies and hemolytic anemias | Idiopathic developmental intellectual disability |
| Arunachal Pradesh | Typhoid and paratyphoid | Conduct disorder | Neonatal disorders | Diarrheal diseases | Dietary iron deficiency | Headache disorders | Asthma | Drowning | Road injuries | Acute hepatitis |
| Delhi | Typhoid and paratyphoid | Dietary iron deficiency | Conduct disorder | Neonatal disorders | Headache disorders | Diarrheal diseases | Road injuries | Dermatitis | Idiopathic epilepsy | Hemoglobinopathies and hemolytic anemias |
| Goa | Conduct disorder | Neonatal disorders | Headache disorders | Idiopathic epilepsy | Drowning | Malaria | Diarrheal diseases | Road injuries | Dermatitis | Dietary iron deficiency |
| Gujarat | Dietary iron deficiency | Typhoid and paratyphoid | Conduct disorder | Neonatal disorders | Diarrheal diseases | Headache disorders | Drowning | Road injuries | Idiopathic epilepsy | Hemoglobinopathies and hemolytic anemias |
| Haryana | Typhoid and paratyphoid | Dietary iron deficiency | Conduct disorder | Neonatal disorders | Road injuries | Diarrheal diseases | Headache disorders | Drowning | Idiopathic epilepsy | Hemoglobinopathies and hemolytic anemias |
| Himachal Pradesh | Neonatal disorders | Conduct disorder | Dietary iron deficiency | Typhoid and paratyphoid | Headache disorders | Diarrheal diseases | Road injuries | Falls | Idiopathic epilepsy | Dermatitis |
| Jammu and Kashmir & Ladakh | Road injuries | Dietary iron deficiency | Conduct disorder | Neonatal disorders | Headache disorders | Typhoid and paratyphoid | Diarrheal diseases | Animal contact | Drowning | Falls |
| Karnataka | Drowning | Dietary iron deficiency | Typhoid and paratyphoid | Conduct disorder | Road injuries | Neonatal disorders | Diarrheal diseases | Headache disorders | Congenital birth defects | Idiopathic epilepsy |
| Kerala | Conduct disorder | Neonatal disorders | Headache disorders | Asthma | Drowning | Road injuries | Dietary iron deficiency | Congenital birth defects | Diarrheal diseases | Dermatitis |
| Maharashtra | Typhoid and paratyphoid | Dietary iron deficiency | Conduct disorder | Neonatal disorders | Headache disorders | Diarrheal diseases | Drowning | Road injuries | Idiopathic epilepsy | Dermatitis |
| Manipur | Typhoid and paratyphoid | Conduct disorder | Neonatal disorders | Diarrheal diseases | Headache disorders | Drowning | Road injuries | Malaria | Idiopathic developmental intellectual disability | Asthma |
| Meghalaya | Malaria | Asthma | Typhoid and paratyphoid | Dietary iron deficiency | Conduct disorder | Neonatal disorders | Diarrheal diseases | Headache disorders | Idiopathic epilepsy | Idiopathic developmental intellectual disability |
| Mizoram | Typhoid and paratyphoid | Malaria | Conduct disorder | Neonatal disorders | Headache disorders | Diarrheal diseases | Road injuries | Dietary iron deficiency | Dermatitis | Drowning |
| Nagaland | Typhoid and paratyphoid | Conduct disorder | Neonatal disorders | Headache disorders | Asthma | HIV/AIDS | Diarrheal diseases | Acute hepatitis | Road injuries | Malaria |
| Other small union territories | Dietary iron deficiency | Conduct disorder | Neonatal disorders | Typhoid and paratyphoid | Headache disorders | Road injuries | Drowning | Diarrheal diseases | Falls | Idiopathic epilepsy |
| Punjab | Typhoid and paratyphoid | Dietary iron deficiency | Conduct disorder | Neonatal disorders | Road injuries | Headache disorders | Diarrheal diseases | Idiopathic epilepsy | Dermatitis | Hemoglobinopathies and hemolytic anemias |
| Sikkim | Typhoid and paratyphoid | Conduct disorder | Neonatal disorders | Diarrheal diseases | Headache disorders | Dietary iron deficiency | Falls | Road injuries | Drowning | Idiopathic epilepsy |
| Tamil Nadu | Dietary iron deficiency | Typhoid and paratyphoid | Drowning | Conduct disorder | Road injuries | Neonatal disorders | Headache disorders | Diarrheal diseases | Congenital birth defects | Falls |
| Telangana | Typhoid and paratyphoid | Dietary iron deficiency | Conduct disorder | Neonatal disorders | Headache disorders | Diarrheal diseases | Road injuries | Drowning | Idiopathic epilepsy | Animal contact |
| Tripura | Malaria | Dietary iron deficiency | Conduct disorder | Neonatal disorders | Diarrheal diseases | Headache disorders | Typhoid and paratyphoid | Drowning | Road injuries | Self-harm |
| West Bengal | Dietary iron deficiency | Conduct disorder | Diarrheal diseases | Neonatal disorders | Headache disorders | Typhoid and paratyphoid | Drowning | Road injuries | Idiopathic developmental intellectual disability | Dermatitis |

**b) 15-19 years**

| **Cause of DALYs** | **Rank 1** | **Rank 2** | **Rank 3** | **Rank 4** | **Rank 5** | **Rank 6** | **Rank 7** | **Rank 8** | **Rank 9** | **Rank 10** |
| --- | --- | --- | --- | --- | --- | --- | --- | --- | --- | --- |
| **India** | **Road injuries** | **Self-harm** | **Headache disorders** | **Typhoid and paratyphoid** | **Diarrheal diseases** | **Neonatal disorders** | **Conduct disorder** | **Dietary iron deficiency** | **Depressive disorders** | **Drowning** |
| **Less developed states** | | | | | | | | | | |
| Assam | Road injuries | Headache disorders | Self-harm | Diarrheal diseases | Cirrhosis and other chronic liver diseases | Typhoid and paratyphoid | Drowning | Neonatal disorders | Tuberculosis | Conduct disorder |
| Bihar | Road injuries | Diarrheal diseases | Headache disorders | Dietary iron deficiency | Typhoid and paratyphoid | Neonatal disorders | Self-harm | Conduct disorder | Depressive disorders | Idiopathic developmental intellectual disability |
| Chhattisgarh | Self-harm | Road injuries | Typhoid and paratyphoid | Headache disorders | Drowning | Interpersonal violence | Malaria | Diarrheal diseases | Dietary iron deficiency | Neonatal disorders |
| Jharkhand | Road injuries | Headache disorders | Diarrheal diseases | Dietary iron deficiency | Conduct disorder | Neonatal disorders | Typhoid and paratyphoid | Depressive disorders | Anxiety disorders | Malaria |
| Madhya Pradesh | Road injuries | Typhoid and paratyphoid | Self-harm | Headache disorders | Diarrheal diseases | Drowning | Dietary iron deficiency | Interpersonal violence | Other unintentional injuries | Tuberculosis |
| Odisha | Road injuries | Self-harm | Headache disorders | Diarrheal diseases | Typhoid and paratyphoid | Malaria | Dietary iron deficiency | Depressive disorders | Neonatal disorders | Idiopathic epilepsy |
| Rajasthan | Road injuries | Typhoid and paratyphoid | Self-harm | Headache disorders | Tuberculosis | Drowning | Diarrheal diseases | Neonatal disorders | Dietary iron deficiency | Conduct disorder |
| Uttar Pradesh | Road injuries | Typhoid and paratyphoid | Self-harm | Headache disorders | Diarrheal diseases | Tuberculosis | Neonatal disorders | Interpersonal violence | Dietary iron deficiency | Conduct disorder |
| Uttarakhand | Road injuries | Typhoid and paratyphoid | Headache disorders | Self-harm | Neonatal disorders | Diarrheal diseases | Drowning | Conduct disorder | Depressive disorders | Idiopathic epilepsy |
| **More developed states** | | | | | | | | | | |
| Andhra Pradesh | Road injuries | Self-harm | Headache disorders | Typhoid and paratyphoid | Depressive disorders | Neonatal disorders | Dietary iron deficiency | Conduct disorder | Diarrheal diseases | Idiopathic epilepsy |
| Arunachal Pradesh | Road injuries | Self-harm | Cirrhosis and other chronic liver diseases | Headache disorders | Neonatal disorders | Diarrheal diseases | Typhoid and paratyphoid | Depressive disorders | Interpersonal violence | Conduct disorder |
| Delhi | Road injuries | Headache disorders | Typhoid and paratyphoid | Neonatal disorders | Self-harm | Conduct disorder | Depressive disorders | Diarrheal diseases | Acne vulgaris | Dietary iron deficiency |
| Goa | Road injuries | Headache disorders | Cirrhosis and other chronic liver diseases | Self-harm | Idiopathic epilepsy | Neonatal disorders | Drowning | Depressive disorders | Conduct disorder | Acne vulgaris |
| Gujarat | Road injuries | Headache disorders | Self-harm | Neonatal disorders | Typhoid and paratyphoid | Conduct disorder | Tuberculosis | Idiopathic epilepsy | Diarrheal diseases | Depressive disorders |
| Haryana | Road injuries | Headache disorders | Typhoid and paratyphoid | Neonatal disorders | Depressive disorders | Self-harm | Conduct disorder | Diarrheal diseases | Acne vulgaris | Idiopathic epilepsy |
| Himachal Pradesh | Road injuries | Headache disorders | Neonatal disorders | Self-harm | Depressive disorders | Conduct disorder | Falls | Typhoid and paratyphoid | Idiopathic epilepsy | Acne vulgaris |
| Jammu and Kashmir & Ladakh | Road injuries | Headache disorders | Neonatal disorders | Self-harm | Conduct disorder | Depressive disorders | Drowning | Dietary iron deficiency | Conflict and terrorism | Falls |
| Karnataka | Self-harm | Road injuries | Headache disorders | Drowning | Neonatal disorders | Idiopathic epilepsy | Depressive disorders | Conduct disorder | Diarrheal diseases | Falls |
| Kerala | Road injuries | Self-harm | Headache disorders | Neonatal disorders | Depressive disorders | Conduct disorder | Asthma | Anxiety disorders | Acne vulgaris | Drowning |
| Maharashtra | Road injuries | Headache disorders | Self-harm | Neonatal disorders | Depressive disorders | Conduct disorder | Typhoid and paratyphoid | Diarrheal diseases | Acne vulgaris | Drowning |
| Manipur | Road injuries | Interpersonal violence | Typhoid and paratyphoid | Self-harm | Headache disorders | Cirrhosis and other chronic liver diseases | Neonatal disorders | Drowning | HIV/AIDS | Depressive disorders |
| Meghalaya | Typhoid and paratyphoid | Asthma | Road injuries | Headache disorders | Self-harm | Malaria | Cirrhosis and other chronic liver diseases | Neonatal disorders | Diarrheal diseases | Depressive disorders |
| Mizoram | Typhoid and paratyphoid | Road injuries | Headache disorders | Neonatal disorders | Self-harm | HIV/AIDS | Conduct disorder | Depressive disorders | Diarrheal diseases | Drowning |
| Nagaland | HIV/AIDS | Road injuries | Typhoid and paratyphoid | Headache disorders | Cirrhosis and other chronic liver diseases | Neonatal disorders | Conduct disorder | Interpersonal violence | Depressive disorders | Tuberculosis |
| Other small union territories | Road injuries | Self-harm | Headache disorders | Neonatal disorders | Depressive disorders | Conduct disorder | Drowning | Cirrhosis and other chronic liver diseases | Falls | Dietary iron deficiency |
| Punjab | Road injuries | Headache disorders | Typhoid and paratyphoid | Neonatal disorders | Conduct disorder | Self-harm | Idiopathic epilepsy | Other unintentional injuries | Ischemic heart disease | Depressive disorders |
| Sikkim | Road injuries | Typhoid and paratyphoid | Headache disorders | Self-harm | Cirrhosis and other chronic liver diseases | Neonatal disorders | Falls | Conduct disorder | Diarrheal diseases | Depressive disorders |
| Tamil Nadu | Road injuries | Self-harm | Headache disorders | Depressive disorders | Neonatal disorders | Drowning | Conduct disorder | Falls | Typhoid and paratyphoid | Dietary iron deficiency |
| Telangana | Road injuries | Self-harm | Headache disorders | Depressive disorders | Neonatal disorders | Typhoid and paratyphoid | Conduct disorder | Idiopathic epilepsy | Diarrheal diseases | Dietary iron deficiency |
| Tripura | Self-harm | Road injuries | Headache disorders | Neonatal disorders | Malaria | Diarrheal diseases | Conduct disorder | Cirrhosis and other chronic liver diseases | Depressive disorders | Typhoid and paratyphoid |
| West Bengal | Road injuries | Self-harm | Headache disorders | Neonatal disorders | Diarrheal diseases | Conduct disorder | Depressive disorders | Dietary iron deficiency | Acne vulgaris | Drowning |

**Table 5. Adolescent health indicators captured in the Adolescent Friendly Health Clinic Management Information System (AFHC MIS).**

| **India Adolescent Health Strategy theme** | **AFHC MIS indicators** | **10-14 years** | **15-19 years** |
| --- | --- | --- | --- |
|  | ***Clinical services received*** |  |  |
| **Improve nutrition** | Iron and folic acid tablets | Data captured for both girls and boys | Data captured for both girls and boys |
|  | Immunization | Data captured for both girls and boys | Data captured for both girls and boys |
| **Enable sexual and reproductive health** | Menstrual problems | Data captured for girls | Data captured for girls |
|  | Reproductive tract infections/sexually transmitted infections management | Data captured for both girls and boys | Data captured for both girls and boys |
|  | Antenatal care | Data captured for girls | Data captured for girls |
|  | Contraceptives |  |  |
|  | *Condoms* | Data captured for both girls and boys | Data captured for both girls and boys |
|  | *Oral contraceptive pills* | Data captured for girls | Data captured for girls |
|  | *Emergency contraceptive pills* | Data captured for girls | Data captured for girls |
|  | *Intrauterine device* | Data captured for girls | Data captured for girls |
| **Non-communicable diseases** | Skin problems | Data captured for both girls and boys | Data captured for both girls and boys |
|  | Any other clinical service offered | Data captured for both girls and boys | Data captured for both girls and boys |
|  | ***Counselling services received*** |  |  |
| **Improve nutrition** | Nutrition | Data captured for both girls and boys | Data captured for both girls and boys |
| **Enable sexual and reproductive health** | Pre-marital counselling | Data captured for both girls and boys | Data captured for both girls and boys |
|  | Sexual problems | Data captured for both girls and boys | Data captured for both girls and boys |
|  | Contraceptive | Data captured for both girls and boys | Data captured for both girls and boys |
|  | Abortion | Data captured for girls | Data captured for girls |
|  | Reproductive tract infections/sexually transmitted infections | Data captured for both girls and boys | Data captured for both girls and boys |
| **Prevent substance misuse** | Substance abuse | Data captured for both girls and boys | Data captured for both girls and boys |
| **Prevent injuries and violence** | Violence | Data captured for both girls and boys | Data captured for both girls and boys |
|  | Sexual abuse | Data captured for both girls and boys | Data captured for both girls and boys |
| **Enhance mental health** | Stress | Data captured for both girls and boys | Data captured for both girls and boys |
|  | Depression | Data captured for both girls and boys | Data captured for both girls and boys |
|  | Suicidal tendency | Data captured for both girls and boys | Data captured for both girls and boys |
|  | Other mental health issues | Data captured for both girls and boys | Data captured for both girls and boys |
| **Non-communicable diseases** | Learning problems | Data captured for both girls and boys | Data captured for both girls and boys |
|  | Skin problems | Data captured for both girls and boys | Data captured for both girls and boys |
|  | Counselling for any other problem | Data captured for both girls and boys | Data captured for both girls and boys |

# **Table 6. Adolescent health indicators available in the Health Management Information System (HMIS).**

|  | **Indicator** | **Age disaggregation** | **Sex disaggregation** |
| --- | --- | --- | --- |
| **Adolescent health** | Number registered in Adolescent Friendly Health Clinics | No | Yes |
|  | Out of those registered, those who received clinical services | No | Yes |
|  | Out of those registered, those who received counselling | No | Yes |
|  | Those in (6th -12th class) provided 4 iron and folic acid tablets in schools | No | Yes |
|  | Those in (6th -12th class) provided albendazole in schools | No | Yes |
|  | Number of out of school adolescent girls (10-19 years) provided 4 iron and folic acid tablets at Anganwadi Centres | No | Data captured for girls |
|  | Number of out of school adolescent girls (10-19 years) provided albendazole at Anganwadi Centres | No | Data captured for girls |
|  | Number of adolescent girls provided sanitary napkin packs | No | Data captured for girls |
|  | Number of sanitary napkin packs sold to adolescent girls | No | Data captured for girls |
|  | Number of sanitary napkin packs distributed free to Accredited Social Health Activist | No | Data captured for girls |
|  | Number of adolescent girls attended monthly meeting | No | Data captured for girls |
| **Details of death reported with probable causes** | Number of adolescent/ adult deaths due to diarrhoeal diseases | No | No |
|  | Number of adolescent/ adult deaths due to tuberculosis | No | No |
|  | Number of adolescent/ adult deaths due to respiratory diseases including infections (other than tuberculosis) | No | No |
|  | Number of adolescent/ adult deaths due to other fever related | No | No |
|  | Number of adolescent/ adult deaths due to HIV/AIDS | No | No |
|  | Number of adolescent/ adult deaths due to heart disease/hypertension related | No | No |
|  | Number of adolescent/ adult deaths due to cancer | No | No |
|  | Number of adolescent/ adult deaths due to neurological disease including stroke | No | No |
|  | Number of adolescent/ adult deaths due to accidents/burn cases | No | No |
|  | Number of adolescent/ adult deaths due to suicide | No | No |
|  | Number of adolescent/ adult deaths due to animal bites and stings | No | No |
|  | Number of adolescent/ adult deaths due to known acute disease | No | No |
|  | Number of adolescent/ adult deaths due to known chronic disease | No | No |
|  | Number of adolescent/ adult deaths due to causes not known | No | No |

|  | Disaggregated data available by age/sex |
| --- | --- |
|  | Disaggregated data not available by age/sex |
